# Data from the Researcher Mental Health Observatory STAIRCASE Survey

**DOI:** 10.5334/jopd.136

**Published:** 2026-05-29

**Authors:** Jana Lasser, Stefan T. Mol, Alja Čontala, Ana Slavec, Andreja Zulim de Swarte, Anna Khachatryan, Anna Maria Eleuteri, Anupoma Haque, Baiba Jansone, Blerina Vrenozi, Brian Cahill, Carlos Trenado, Christiane Schwieren, Claudia Iuliana Iacob, Claudia Tejada-Gallardo, Cornelia Mairean, Darragh McCashin, Deborah Chery, Dilara Özel, Dimity Stephen, Dragan Mijakoski, Elena Ronda, Eleonora Ricci, Eliana Ibrahimi, Emese Vita, Fatjona Kamberi, Fernando G. Benavides, Francisco Valente Gonçalves, Gábor Kismihók, Gemma Pascual Manich, Igor Esnaola, Igor Portoghese, Inge van der Weijden, Irina Guseva Canu, Irsida Mehmeti, Ivana B. Petrovic, Jakub Šindelář, João Miguel Alves Ferreira, Jonila Gabrani, Karolina Eszter Kovács, Katri Pöllänen, Konstantinos Geles, Lamis Yahia Mohamed Elkheir, Lorena Cudris-Torres, Lise T. Løvseth, Louiza Ioannidou, Luksa Popovic, Mais M. Aljunaidy, Maria Fatjó Saboya, Marit Christensen, Marlene Santos, Marta Miklikowska, Mateja Erce Paoli, Mathias Schroijen, Mathieu Fusi, Mayya Sundukova, Mete Kurtoğlu, Milica Vukelić, Mirela Adriana Anghelache, Mohamad Nadim Adi, Mümine Barkçin, Nuno Barbosa Rocha, Nurka Pranjic, Olga Bogolyubova, Paulo Alexandre Soares Moreira, Paulo Moreira, Pil Maria Saugmann, Rivka Tuval-Mashiach, Rui Amaral Mendes, Sabina Osmanovic, Sinem Ozdede, Simone Lackner, Stéphanie Gauttier, Szidónia Rusu, Theodota Lagouri, Ümran Yüce-Selvi, Urszula Ziemiańczyk, Xhilda Dhamo

**Affiliations:** 1 IDea_Lab, University of Graz, AT; 2 Amsterdam Business School, University of Amsterdam, NL; 3 Advanced Materials Department, Jožef Stefan Institute and Jožef Stefan International Postgraduate School and Department of Chemistry and Biochemistry, The University of Toledo, US; 4 Dr. Nina McClelland Laboratory for Water Chemistry and Environmental Analysis, Department of Chemistry and Biochemistry, The University of Toledo, US; 5 Faculty of Mathematics, Natural Sciences and Information Technologies, University of Primorska, SI; 6 InnoRenew CoE Department, Andrej MarušičInstitute, University of Primorska, SI; 7 University Medical Centre Utrecht, NL; 8 Independent Researcher; 9 Department of Biosciences and Veterinary Medicine, University of Camerino, IT; 10 ProRare Austria, Austria; 11 Department of Neuromedicine and Neurosciences, Faculty of Medicine and Life Sciences, University of Latvia, LV; 12 Research Center of Flora and Fauna, University of Tirana, AL; 13 Bielefeld University of Applied Sciences, DE; 14 Leibniz Information Centre for Science and Technology, DE; 15 Institute of Clinical Neuroscience and Medical Psychology, Heinrich Heine University Düsseldorf, DE; 16 Max Planck Institute for Empirical Aesthetics, DE; 17 Alfred Weber Institute of Economics, Heidelberg University, DE; 18 Department of Applied Psychology and Psychotherapy, University of Bucharest, RO; 19 Department of Psychology, Sociology, and Social Work, Universitat de Lleida, ES; 20 Department of Psychology, Alexandru Ioan Cuza University of Iasi, RO; 21 School of Psychology, Dublin City University, IE; 22 Lycée Victor Schoelcher Rectorat de Martinique Direction des Personnels Les hauts de Terreville, FR; 23 School of Education, University of Glasgow, UK; 24 German Centre for Higher Education Research and Science Studies, Berlin, DE; 25 Institute of Occupational Health of R.N. Macedonia and Faculty of Medicine, Ss. Cyril and Methodius, University in Skopje, Skopje, MK; 26 Public Health Department, University of Alicante, ES; 27 University of Edinburgh, Edinburgh, UK; 28 National Centre for Scientific Research “Demokritos”, Athens, GR; 29 Department of Biology, University of Tirana, AL; 30 Scientific Research Centre for Public Health, University of Vlore “Ismail Qemali”, AL; 31 CiSAL-Centro de Investigación en Salud Laboral, Universidad Pompeu Fabra, ES; 32 Hospital del Mar Research Institute and CIBER de Epidemiología y Salud Pública, Instituto de Salud Carlos III, ES; 33 RUMO.Solutions, PT; 34 Faculty of Health Sciences, Universidade Europeia, PT; 35 TIB –Leibniz Information Centre for Science and Technology, DE; 36 Institut d’Investigacions Biomèdiques August Pi i Sunyer, ES; 37 Universidad del País Vasco/Euskal Herriko Unibertsitatea, UPV/EHU, ES; 38 University of Cagliari, IT; 39 Centre for Science and Technology Studies, Leiden University, NL; 40 Department of Occupational and Environmental Health, Unisanté, University of Lausanne, CH; 41 Catholic University Our Lady of Good Counsel, AL; 42 Department of Psychology, University of Belgrade, RS; 43 Charles University, CZ; 44 Leiden University, NL; 45 Czech Association of Doctoral Researchers, CZ; 46 Institute of Pharmacology and Experimental Therapeutics, PT; 47 Coimbra Institute for Clinical and Biomedical Research, University Coimbra, PT; 48 Center for Innovative Biomedicine and Biotechnology, University Coimbra, PT; 49 European University of Tirana, AL; 50 University of Debrecen, HU; 51 Department of Cultures, University of Helsinki, FI; 52 Department of Pharmaceutical Chemistry, Faculty of Pharmacy, University of Khartoum, SD; 53 African Reproducibility Network, GH; 54 Department of Social Sciences, Universidad de la Costa, CO; 55 Clinic of Mental Health, St Olav University Hospital of Trondheim, NO; 56 European University Cyprus, CY; 57 Faculty of Science, University of Split, HR; 58 Department of Psychology, Texas State University, San Marcos, TX, US; 59 Department of Psychology, Norwegian University of Science and Technology, NO; 60 REQUIMTE/LAQV, Escola Superior de Saúde, Instituto Politécnico do Porto, PT; 61 Molecular Oncology & Viral Pathology, IPO-Porto Research Center, Portuguese Institute of Oncology, PT; 62 Institute for Globally Distributed Open Research and Education, SE; 63 Linnaeus University, SE; 64 Universitélibre de Bruxelles, BE; 65 Instituto Investigación Sanitaria Biobizkaia, ES; 66 Middle East Technical University, TR; 67 Institute of Geodynamics of Romanian Academy, RO; 68 Department of Interior Design, Texas State University, San Marcos, TX, US; 69 Hacettepe University, TR; 70 RISE-Health, Center for Translational Health, PT; 71 Medical Biotechnology Research, E2S, Polytechnic of Porto, PT; 72 Faculty of Medicine, University of Tuzla, BA; 73 Institute of Security and Global Affairs, Leiden University, The Hague, NL; 74 University of Trás-os-Montes and Alto Douro and CIDESD, PT; 75 Atlantica Instituto Universitario, Oeiras, PT; 76 International Healthcare Management Research and Development Centre at the First Affiliated Hospital of Shandong First Medical University and Shandong Provincial Qianfoshan Hospital, PT; 77 Eurodoc, BE; 78 Bar ilan University, IL; 79 MEDCIDS –Department of Community Medicine, Information and Health Decision Sciences, Faculty of Medicine, University of Porto, PT; 80 RISE-Health, Faculty of Medicine, University of Porto, PT; 81 Case Western Reserve University, US; 82 University of Montenegro, ME; 83 Department of Architecture and Design, University of Pamukkale, Denizli, TR; 84 Grenoble Ecole de Management, FR; 85 SciLink Foundation, NL; 86 Babeș-Bolyai University, RO; 87 Marie Curie Alumni Association, FR; 88 Department of Psychology, Eskisehir Osmangazi University, TR; 89 University of Agriculture in Krakow, PL; 90 Department of Applied Mathematics, Faculty of Natural Sciences, University of Tirana, AL

**Keywords:** mental health, academia, researchers, working conditions, occupational health

## Abstract

The data presented here derives from the Sustainable Working Conditions in Academia survey (STAIRCASE) on researcher mental health. The survey addresses the ongoing mental health crisis in academia by providing comprehensive, multilevel data on researcher well-being and its structural determinants. We employed a cross-sectional between-participant study design to collect data from 4,296 researchers between September 15, 2023, and August 26, 2024. The sample, which primarily includes researchers based in European countries, has a mean age of 38.1 years (SD = 10.7) and consists of 63.7% female participants. Participants provided data on key mental health outcomes – including depression, anxiety, stress, burnout, and well-being – alongside detailed assessments of working conditions, leadership behavior, and organizational characteristics. The dataset facilitates a holistic, multilevel investigation of academic mental health beyond individual risk factors by including the individual, leadership, institutional, and national context. By enabling analyses across hierarchical levels this dataset provides the necessary evidence to identify systemic drivers of mental (ill) health and inform the development of effective, system-wide strategies for meaningful change in academic work environments.

## (1) Background

Researchers play a vital role in producing knowledge aimed at finding solutions, expanding knowledge and advancing our understanding in all disciplines and sectors. Worryingly, a growing body of research suggests that researchers at all career levels exhibit high levels of stress, anxiety, depression, and burnout (Hazell et al., 2020; Levecque et al., 2017; Nicholls et al., 2022; Urbina-Garcia, 2020), signalling that academia is contending with a mental health crisis. More specifically, Hazell, et al. (2020) based on a meta-analysis of nine US studies reporting Perceived Stress Scale (PSS) data of doctoral researchers report a large and significant between-group effect size relative to PSS scores of the general population. In the European context, Levecque et al., in their discipline and university representative study of PhD candidates in Flanders, Belgium, similarly report that the prevalence of them having or being likely to develop a common psychiatric disorder is at least 1.5 times as high when compared to higher education groups, and nearly two times as high as that of the general population. And although likely more prevalent at early career levels, due to issues associated with precarity and the prevalence of top-down power dynamics that serve to enshrine silence around instances of bullying, harassment, and exploitation (Nicholls et al., 2022), there is evidence of an elevated prevalence of mental health issues at higher ranks as well. Indeed, Urbina-Garcia (2020), based on a systematic literature review of academics’ mental health, concludes that across studies conducted around the world academic staff experience high stress and burnout and poor well-being.

Although a significant portion of the literature has approached academic mental health at the level of individual resources and risk factors, this approach risks missing the larger, structural picture. In fact, researchers face numerous stressors (or job demands), including job insecurity, financial difficulties, a unrealistically high workloads, and lack of support, which are increasingly being identified as primary determinants of deteriorating mental health outcomes (Bal et al., 2019; Evans et al., 2018; Lau & Pretorius, 2019). Perverse incentives in academia that focus on competition rather than rigour, transparency and collaboration, particularly in a context with reduced (and oftentimes diminishing) job resources, may further exacerbate the problem, potentially compromising research integrity and undermining the research enterprise’s capacity to address global challenges (Edwards & Roy, 2017). The replication crisis in several fields and scientific fraud scandals are a case in point.

The existing literature referenced above is skewed toward analyses of individual resources and risk factors rather than examining the broader context, a pattern that might be rooted in a reluctance to critique systems that support researchers, misalignment with reward structures emphasizing quantity over quality of work, and the complexities involved in conducting the multilevel research that is needed to establish contextual effects. Furthermore, interventions targeting individuals might seem faster and cheaper than those targeting structures. However, the factors contributing to individual mental (ill) health outcomes are likely to reside at varying hierarchical levels in the academic system, from the individual to the research group and department, to the institution, and even the country level. In addition, the dual factor model of mental health (Keyes, 2005; Westerhof & Keyes, 2010) and research (Huppert, 2009) suggests that the drivers of mental (ill) health are not the same as those of well-being. Therefore, a more comprehensive approach is needed to address the academic mental health crisis, one that considers not only individual traits but also the substantial influence of contextual elements, such as leadership, work culture, institutional policies, and societal expectations. Here, we operationalise mental health in terms of both diagnosable symptoms such as anxiety, stress, and depression, but also in terms of broader aspects of well-being – such as a sense of purpose, satisfaction, and the ability to function effectively in one’s professional and personal life, measured using standardized psychometric scales. This holistic perspective is expected to provide a more comprehensive understanding of the aforementioned crisis, with which in turn we hope to inform more effective and system-wide strategies for improving mental health in academia.

In sum, the STAIRCASE (Sustainable Working Conditions in Academia) Survey aims to broaden our understanding of the impact of working conditions on mental health in academia, yielding evidence-based recommendations for impactful change. The present data set is the result of the first round of data collected with this survey that aimed to assess the mental health of researchers at all career levels across European countries, as well as their working conditions, the characteristics of their workplaces, and the characteristics of the leadership behaviour of their superiors. The survey is part of a larger endeavour that aims to assess and improve mental health of researchers, organised under the umbrella of the EU Funded COST Action CA19117 “Researcher Mental Health Observatory” (ReMO).

The ReMO consortium aims to facilitate the study of researcher mental health and its antecedents by providing access to all data collected in the most transparent and accessible way possible, while respecting data protection requirements under the General Data Protection Regulation (GDPR). To this end, the data collected via the STAIRCASE survey is made accessible via a Campus Use File (CUF) that has been fully anonymised and is accessible for download and is described in this article. In addition, we provide a Scientific Use File (SUF) that is accessible to researchers only after entering into a Data Use Agreement and in a secure analysis environment subject to output controls. Information specific to the SUF can be found in the Appendix.

## (2) Methods

### 2.1 Study design

The study follows a cross-sectional design comparing different groups and allowing for a multilevel statistical analysis. Measures were selected to inform constructs at each level of the Individual, Group, Leader, Organisation, and the Outside Context (IGLOO) model that considers mental health in the workplace as the interaction of resources at the individual, group, leader and organisational levels (Nielsen et al., 2018). We add information about the country to this perspective, as we suspect variance on this level due to, among other factors, different research cultures, policies and legal frameworks, and funding conditions. It should be noted that the envisaged multilevel analysis only comprises individual, organization, and country level data due to privacy concerns associated with having identifiable departments and leaders. On the other hand, the research field could be used as one level of analysis, since it likely plays a crucial role in defining work culture. Data was collected via an online survey administered through LimeSurvey between September 2023 and August 2024. In this manuscript, we provide an overview of the central foci of the survey. Details and sources for each measurement instrument are provided in Section 2.5. The number of respondents collected from different countries is listed in Table 1.

**Table 1:** Number of responses per country with at least 24 respondents.


COUNTRY	N RESPONSES	% OF RESEARCH POPULATION SAMPLED	% FEMALE RESPONSES	% FEMALE IN RESEARCH POPULATION

Albania	48	11.26	79.2	44.3

Austria	50	0.08	58.0	31.3

Belgium	88	0.11	70.5	33.5

Switzerland	741	1.38	65.0	37.5

Czech Republic	80	0.16	56.3	27.7

Germany	161	0.03	54.0	29.4

Spain	305	0.19	61.6	41.6

Finland	100	0.22	58.0	33.6

France	585	0.16	54.2	29.7

Great Britain	32	0.01	65.6	38.7

Greece	116	0.23	55.2	40.1

Hungary	56	0.13	64.9	28.8

ireland	51	0.17	78.4	37.4

Italy	168	0.10	53.6	36.4

Latvia	69	1.62	68.1	50.7

North Macedonia	304	18.03	65.8	54.3

Malta	43	3.20	74.4	35.7

Montenegro	93	19.74	63.4	52.1

The Netherlands	63	0.06	69.8	30.2

Portugal	137	0.23	73.7	42.1

Romania	103	0.54	68.0	46.1

Serbia	27	0.16	66.7	53.5

Slovakia	120	0.65	64.2	40.0

Slovenia	210	1.84	62.4	35.0

Sweden	33	0.04	69.7	34.6

Turkey	102	0.05	70.6	37.3


Note: For countries with >24 responses, the number of responses is divided by the total number of researchers in each country based on the latest UNESCO indicator “Researchers per million inhabitants (FTE)”^1^ available for the country to calculate the percentage of researchers in the country that responded to the survey. The column “% female responses” is the number of responses from respondents who reported to be female divided by the total number of responses from that country. The column “% female in research population” is the percentage of female researchers in each country based on the latest UNESCO indicator “Researchers (HC) – % Female”^2^ available for the country.

The survey encompassed items for demographic data, including information specific to the research profession, such as the research field and the time since completion of the PhD degree (see Table 2 for a list of all assessed demographic variables). The main outcomes the survey aimed to measure using standardized and validated psychometric scales were work engagement, burnout, stress, anxiety, depression, and well-being, as well as resilience as a personal resource (see Table 3 for a list of all assessed constructs related to health and well-being of respondents). In addition, the survey included a number of items to assess the working and studying conditions, including the characteristics of the work contract, time spent on different tasks and time allocated for professional development (see Table 4 for a list of all assessed variables related to working and studying conditions). The working and studying social environment was also assessed via several standard questionnaires, including job predictability, role clarity, policy perceptions, job insecurity, job control, interpersonal conflict, work-family conflict, sense of community, illegitimate tasks, work-related stress, emotional support, instrumental support, and job satisfaction (see Table 5 for a list of all assessed variables related to the working and social environment). Respondents were also asked whether they experienced harassment or observed harassment of a coworker, what role the perpetrator of the harassment had and the (perceived) basis for the harassment (see Table 6 for a list of all assessed variables). Lastly, the characteristics of the respondent’s perception of their leader and supervisor (if applicable) were assessed using validated questionnaires, as well as the number of meetings with the supervisor (see Table 7 for a list of all assessed constructs).

**Table 2:** Variables in the “Demographics” section of the survey.


VARIABLE CONTENT	VARIABLE LABEL	% MISSING	CUF PROCESSING

Participant ID	participant_id	0.0	Deleted and newly randomised.

Primary position	primary_position	6.3	Merged R3 “established researcher” and R4 “leading researcher” categories.

Gender	gender	6.9	“non binary” set to NA

Age	age	5.4	Calculated age from year of birth. Summarised into <31 years, 31–42 years, >42 years.

Nationality	nationality	12.2	Deleted

Nationality region	nationality_region	6.9	Deleted

Nationality continent	nationality_continent	5.8	Deleted

Place of work country	country_of_work	9.0	Deleted

Place of work region	country_of_work_region	5.4	Deleted

Place of work continent	country_of_work_continent	5.4	Derived from country_of_work_region using UN region coding. Summarised into “europe” and “outside europe”

Research field	research_field	6.4	Mapped free-text answers to existing categories. Summarised into “Chemistry, Physics, Mathematics”, “Engineering and Information Science; Environment or Geo-Science”, “Social and Human Sciences; Economics”

Research field – subfield	NA	NA	Deleted

Civil status	civil_status	7.9	Mapped free-text answers to existing categories. Summarised into “in a relationship/married” and “single”.

Partner work	partner_work	32.4	Deleted

Babies in household	babies_in_household	7.2	Derived from a question asking for the number of babies by mapping 0 to “no” and numbers >0 to “yes”.

Children in household	children_in_household	7.0	Derived from a question asking for the number of children by mapping 0 to “no” and numbers >0 to “yes”.

Adolescents in household	adolescents_in_household	7.1	Derived from a question asking for the number of adolescents by mapping 0 to “no” and numbers >0 to “yes”.

Young adults in household	young_adults_in_household	7.0	Derived from a question asking for the number of young adults by mapping 0 to “no” and numbers >0 to “yes”.

Income	income	5.7	

Time since PhD degree	years_since_phd_completion	50.3	Calculated age from year of PhD completion. Summarised into 1–15 years and >15 years.


Note: Variable content, variable label in the data set, number of missing responses – e.g. where respondents chose “I prefer not to say” or “Does not apply” (for the “Time since PhD degree” question), chose to terminate the survey before reaching the given question, or were not shown a question because of a filter (for the “Partner work” question) –, and processing steps for the CUF data file for variables in the “Demographics” section of the survey.

**Table 3:** Variables in the “Mental health outcomes” section of the survey.


VARIABLE CONTENT	VARIABLE LABEL	% MISSING	RESPONSE SCALE	RESPONSE SCALE NUMERIC

Work-engagement item 1	UWES_1	13.3	Never, Rarely, Sometimes, Often, Very often, Always	1, 2, 3, 4, 5, 6

Work-engagement item 2	UWES_2	13.3	see above	1, 2, 3, 4, 5, 6

Work-engagement item 3	UWES_3	13.8	see above	1, 2, 3, 4, 5, 6

Burnout item 1	BAT_1	13.3	Never, Rarely, Sometimes, Often, Always	1, 2, 3, 4, 5

Burnout item 2	BAT_2	13.3	see above	1, 2, 3, 4, 5

Burnout item 3	BAT_3	13.3	see above	1, 2, 3, 4, 5

Burnout item 4	BAT_4	13.4	see above	1, 2, 3, 4, 5

Burnout item 5	BAT_5	14.0	see above	1, 2, 3, 4, 5

Burnout item 6	BAT_6	14.8	see above	1, 2, 3, 4, 5

Burnout item 7	BAT_7	13.4	see above	1, 2, 3, 4, 5

Burnout item 8	BAT_8	13.4	see above	1, 2, 3, 4, 5

Burnout item 9	BAT_9	13.4	see above	1, 2, 3, 4, 5

Burnout item 10	BAT_10	13.9	see above	1, 2, 3, 4, 5

Burnout item 11	BAT_11	13.5	see above	1, 2, 3, 4, 5

Burnout item 12	BAT_12	13.4	see above	1, 2, 3, 4, 5

Resilience item 1	BRS_1	14.1	Strongly disagree, Disagree, Neutral, Agree, Strongly agree	1, 2, 3, 4, 5

Resilience item 2	BRS_2	13.7	see above	5, 4, 3, 2, 1

Resilience item 3	BRS_3	13.6	see above	1, 2, 3, 4, 5

Resilience item 4	BRS_4	14.1	see above	5, 4, 3, 2, 1

Resilience item 5	BRS_5	13.8	see above	1, 2, 3, 4, 5

Resilience item 6	BRS_6	14.2	see above	5, 4, 3, 2, 1

Stress and anxiety item 1	DASS_1	15.3	Did not apply to me at all, Applied to me to some degree, Applied to me to a considerable degree, or a good part of the time, Applied to me very much or most of the time	0, 1, 2, 3

Stress and anxiety item 2	DASS_2	15.0	see above	0, 1, 2, 3

Stress and anxiety item 3	DASS_3	14.4	see above	0, 1, 2, 3

Stress and anxiety item 4	DASS_4	14.2	see above	0, 1, 2, 3

Stress and anxiety item 5	DASS_5	14.2	see above	0, 1, 2, 3

Stress and anxiety item 6	DASS_6	14.2	see above	0, 1, 2, 3

Stress and anxiety item 7	DASS_7	14.2	see above	0, 1, 2, 3

Stress and anxiety item 8	DASS_8	14.3	see above	0, 1, 2, 3

Stress and anxiety item 9	DASS_9	14.2	see above	0, 1, 2, 3

Stress and anxiety item 10	DASS_10	14.3	see above	0, 1, 2, 3

Stress and anxiety item 11	DASS_11	14.6	see above	0, 1, 2, 3

Stress and anxiety item 12	DASS_12	14.2	see above	0, 1, 2, 3

Stress and anxiety item 13	DASS_13	14.5	see above	0, 1, 2, 3

Stress and anxiety item 14	DASS_14	15.6	see above	0, 1, 2, 3

Stress and anxiety item 15	DASS_15	14.2	see above	0, 1, 2, 3

Stress and anxiety item 16	DASS_16	14.2	see above	0, 1, 2, 3

Stress and anxiety item 17	DASS_17	14.4	see above	0, 1, 2, 3

Stress and anxiety item 18	DASS_18	15.0	see above	0, 1, 2, 3

Stress and anxiety item 19	DASS_19	14.6	see above	0, 1, 2, 3

Stress and anxiety item 20	DASS_20	14.3	see above	0, 1, 2, 3

Stress and anxiety item 21	DASS_21	14.6	see above	0, 1, 2, 3

Well-being item 1	WHO-5_1	13.9	All of the time, Most of the time, More than half of the time, Less than half of the time, Some of the time, At no time	5, 4, 3, 2, 1, 0

Well-being item 2	WHO-5_2	13.8	see above	5, 4, 3, 2, 1, 0

Well-being item 3	WHO-5_3	13.9	see above	5, 4, 3, 2, 1, 0

Well-being item 4	WHO-5_4	13.9	see above	5, 4, 3, 2, 1, 0

Well-being item 5	WHO-5_5	14.0	see above	5, 4, 3, 2, 1, 0


Note: Variable content, variable label in the data set, number of missing responses – e.g. where respondents chose “I prefer not to say” or chose to terminate the survey before reaching the given question –, answer options on scales, and numerical mapping of the answer options in the data files for variables in the “Mental health outcomes” section (e.g. outcomes) of the survey.

**Table 4:** Variables in the “Working- and studying conditions” section of the survey.


VARIABLE CONTENT	VARIABLE LABEL	% MISSING	CUF PROCESSING

Main place of work	main_place_of_work_hashed	33.9	Deleted

Additional places of work	other_work	17.9	

Contract duration	contract_duration	20.6	The original question provided the answer option “I have a tenure-track position” that was non-exclusive with answer options “Within the next 12 months”, “In 1–2 years” “In 2–3 years”, and “In more than 3 years”. This answer was transformed into a separate column. The remaining answers were summarised into “permanent position” and “termed contract/no contract/stipend”.

Tenure track	tenure_track	1.9	Created by mapping the response “I have a tenure track position” from the contract duration question (see above) to “yes” and all other responses to “no”.

Overwork	working_hours_overtime	39.6	Created by subtracting the answer to the question “How many working hours per week are specified in your contract or funding for your main place of work?” from the answer to the question “How many hours per week do you actually work (on average)?”

Time spent on teaching	percent_teaching	45.3	Deleted

Time spent on research	percent_research	18.0	Deleted

Time spent on applying for research funding	percent_funding	54.6	Deleted

Time spent on mentoring	percent_mentoring	46.7	Deleted

Time spent on administrative tasks	percent_admin	27.1	Deleted

Time spent on other tasks	percent_other	51.6	Deleted

Research output	research_output	0.0	The original question asked for the number of research outputs in the categories “Peer reviewed journal article”, “Peer reviewed conference article”, “Monograph”, “Patent” and “Other”. The variable was created by adding the numbers provided for each of the five output subcategories. Not providing a number defaulted to 0, there was no “I prefer not to say” answer option. The number was then summarised into 0, 1–5 and >5.

Professional development	days_professional_development	18.7	

Leaving academia	leaving_academia	18.3	

Recommending academia	recommend_academia	18.3	


Note: Variable content, variable label in the data set, number of missing responses – e.g. where respondents chose “I prefer not to say”, chose to terminate the survey before reaching the given question, or did not enter a number (the “Time spent on task” questions) –, and processing steps for the CUF data file for variables in the “Working- and studying conditions” section of the survey.

**Table 5:** Variables in the “Working- and studying environment” section of the survey.


VARIABLE CONTENT	VARIABLE LABEL	% MISSING	RESPONSE SCALE	RESPONSE SCALE NUMERIC

Predictability item 1	COPSOQ_predictability_1	23.9	To a very large extent, To a large extent, To some extent, To a little extent, To a very little extent	100, 75, 50, 25, 0

Predictability item 2	COPSOQ_predictability_2	23.6	see above	100, 75, 50, 25, 0

Predictability item 3	COPSOQ_predictability_3	24.1	see above	0, 25, 50, 75, 100

Role clarity item 1	role_clarity_1	25.2	To a very large extent, To a large extent, To some extent, To a little extent, To a very little extent	100, 75, 50, 25, 0

Role clarity item 2	role_clarity_2	23.6	see above	100, 75, 50, 25, 0

Role clarity item 3	role_clarity_3	23.6	see above	100, 75, 50, 25, 0

Role clarity item 4	role_clarity_4	23.5	see above	100, 75, 50, 25, 0

Sense of community item 1	COPSOQ_sense_of_community_1	23.9	Hardly ever/almost never, Seldom, Sometimes, Often, Extremely often/always	0, 25, 50, 75, 100

Sense of community item 2	COPSOQ_sense_of_community_2	23.9	see above	0, 25, 50, 75, 100

Sense of community item 3	COPSOQ_sense_of_community_3	24.0	see above	0, 25, 50, 75, 100

Emotional support item 1	COPSOQ_emotional_support_1	32.0	Hardly ever/almost never, Seldom, Sometimes, Often, Extremely often/always	0, 25, 50, 75, 100

Emotional support item 2	COPSOQ_emotional_support_2	36.1	see above	0, 25, 50, 75, 100

Emotional support item 3	COPSOQ_emotional_support_3	26.0	see above	0, 25, 50, 75, 100

Emotional support item 4	COPSOQ_emotional_support_4	37.5	see above	0, 25, 50, 75, 100

Emotional support item 5	COPSOQ_emotional_support_5	25.4	see above	0, 25, 50, 75, 100

Instrumental support item 1	COPSOQ_instrumental_support_1	24.9	Hardly ever/almost never, Seldom, Sometimes, Often, Extremely often/always	0, 25, 50, 75, 100

Instrumental support item 2	COPSOQ_instrumental_support_2	25.6	see above	0, 25, 50, 75, 100

Instrumental support item 3	COPSOQ_instrumental_support_3	24.3	see above	0, 25, 50, 75, 100

Instrumental support item 4	COPSOQ_instrumental_support_4	25.3	see above	0, 25, 50, 75, 100

Instrumental support item 5	COPSOQ_instrumental_support_5	24.3	see above	0, 25, 50, 75, 100

Policy perceptions item 1	policy_perceptions_1	27.2	To a very large extent, To a large extent, To some extent, To a little extent, To a very little extent	5, 4, 3, 2, 1

Policy perceptions item 2	policy_perceptions_2	28.5	see above	5, 4, 3, 2, 1

Policy perceptions item 3	policy_perceptions_3	27.0	see above	5, 4, 3, 2, 1

Policy perceptions item 4	policy_perceptions_4	29.4	see above	5, 4, 3, 2, 1

Policy perceptions item 5	policy_perceptions_5	42.3	see above	5, 4, 3, 2, 1

Policy perceptions item 6	policy_perceptions_6	42.5	see above	5, 4, 3, 2, 1

Policy perceptions item 7	policy_perceptions_7	29.0	see above	5, 4, 3, 2, 1

Job insecurity item 1	job_insecurity_1	25.6	Fully disagree, Somewhat disagree, Neither disagree/nor agree, Somewhat agree, Fully agree	5, 4, 3, 2, 1

Job insecurity item 2	job_insecurity_2	25.7	see above	1, 2, 3, 4, 5

Job insecurity item 3	job_insecurity_3	25.0	see above	5, 4, 3, 2, 1

Job insecurity item 4	job_insecurity_4	26.1	see above	5, 4, 3, 2, 1

Job control item 1	job_control_1	23.8	Fully disagree, Somewhat disagree, Neither disagree/nor agree, Somewhat agree, Fully agree	1, 2, 3, 4, 5

Job control item 2	job_control_2	23.7	see above	1, 2, 3, 4, 5

Job control item 3	job_control_3	23.6	see above	1, 2, 3, 4, 5

Interpersonal conflict item 1	interpersonal_conflict_1	25.1	Fully disagree, Somewhat disagree, Neither disagree/nor agree, Somewhat agree, Fully agree	1, 2, 3, 4, 5

Interpersonal conflict item 2	interpersonal_conflict_2	25.4	see above	1, 2, 3, 4, 5

Interpersonal conflict item 3	interpersonal_conflict_3	24.8	see above	1, 2, 3, 4, 5

Work-family reconciliation item 1	work_family_recon_1	24.0	Fully disagree, Somewhat disagree, Neither disagree/nor agree, Somewhat agree, Fully agree	1, 2, 3, 4, 5

Work-family reconciliation item 2	work_family_recon_2	25.0	see above	1, 2, 3, 4, 5

Work-family reconciliation item 3	work_family_recon_3	24.1	see above	1, 2, 3, 4, 5

Work-family reconciliation item 4	work_family_recon_4	25.2	see above	1, 2, 3, 4, 5

Work-family reconciliation item 5	work_family_recon_5	24.6	see above	1, 2, 3, 4, 5

Illegitimate tasks item 1	illegitimate_tasks_1	24.2	Hardly ever/almost never, Seldom, Sometimes, Often, Extremely often/always	1, 2, 3, 4, 5

Illegitimate tasks item 2	illegitimate_tasks_2	24.7	see above	1, 2, 3, 4, 5

Illegitimate tasks item 3	illegitimate_tasks_3	24.5	see above	1, 2, 3, 4, 5

Illegitimate tasks item 4	illegitimate_tasks_4	24.6	see above	1, 2, 3, 4, 5

Work-related stress item 1	work_related_stress_1	24.1	Hardly ever/almost never, Seldom, Sometimes, Often, Extremely often/always	1, 2, 3, 4, 5

Work-related stress item 2	work_related_stress_2	23.8	see above	1, 2, 3, 4, 5

Work-related stress item 3	work_related_stress_3	24.1	see above	1, 2, 3, 4, 5

Job satisfaction item 1	job_satisfaction	23.8	0, 1, 2, 3, 4, 5, 6, 7, 8, 9, 10	0, 1, 2, 3, 4, 5, 6, 7, 8, 9, 10


Note: Variable content, variable label in the data set, number of missing responses – e.g. where respondents chose “I prefer not to say” or chose to terminate the survey before reaching the given question –, answer options on scales, and numerical mapping of the answer options in the data files for variables in the “Working- and studying environment” section of the survey.

**Table 6:** Variables in the “Working- and studying environment – harassment” subsection of the survey.


VARIABLE CONTENT	VARIABLE LABEL	% MISSING	CUF PROCESSING

Perceived harassment	harassment_perceived	28.2	

Perpetrator of perceived harassment supervisor	harassment_perceived_perp_supervisor	0.0	Derived from a question about the perpetrator of perceived harassment that allowed for selecting more than one answer option. “I prefer not to say” was coded as a separate category and not mapped to NA.The variable is set to “True” if the supervisor was selected and to “False” if the supervisor was not selected.

Perpetrator of perceived harassment coworker	harassment_perceived_perp_coworker	0.0	… if a coworker was selected.

Perpetrator of perceived harassment administrative personnel	harassment_perceived_perp_administration	0.0	… if administrative personnel was reported. Analysis of the free-text answers to this question yielded a high number of answers indicating administrative personnel as perpetrators in perceived harassment, warranting the creation of the category that was coded from the free-text answers.

Perpetrator of perceived harassment student	harassment_perceived_perp_student	0.0	… if a student was selected. Analysis of the free-text answers to this question yielded a high number of answers indicating students as perpetrators in perceived harassment, warranting the creation of the category that was coded from the free-text answers.

Perpetrator of perceived harassment prefer not to say	harassment_perceived_perp_pns	0.0	… if “I prefer not to say” was selected.

Reason of perceived harassment gender	harassment_perceived_reason_gender	0.0	Derived from a question about the reason for the perceived harassment that allowed for selecting more than one answer option. The variable is set to “True” if gender was selected and to “False” if gender was not selected.

Reason of perceived harassment sexual orientation	harassment_perceived_reason_sexorient	0.0	… if sexual orientation was selected.

Reason of perceived harassment religion	harassment_perceived_reason_religion	0.0	… if religion was selected.

Reason of perceived harassment ethnicity	harassment_perceived_reason_ethnicity	0.0	… if ethnicity was selected.

Reason of perceived harassment culture	harassment_perceived_reason_culture	0.0	… if culture was selected.

Reason of perceived harassment hierarchy	harassment_perceived_reason_hierarchiy	0.0	… if hierarchy was selected.

Reason of perceived harassment age	harassment_perceived_reason_age	0.0	… if age was selected.

Reason of perceived harassment disability	harassment_perceived_reason_disability	0.0	… if disability was selected.

Reason of perceived harassment other	harassment_perceived_reason_other	96.5	The variable contains the anonymised free-text answers that respondents were asked to provide if “other” was selected.

Reason of perceived harassment don’t know	harassment_perceived_reason_idk	0.0	… if “I don’t know” was selected.

Reason of perceived harassment prefer not to say	harassment_perceived_reason_pns	0.0	… if “I prefer not to say” was selected.

Experienced harassment	harassment_experienced	26.8	

Perpetrator of experienced harassment supervisor	harassment_experienced_perp_supervisor	0.0	Derived from a question about the perpetrator of experienced harassment that allowed for selecting more than one answer option. “I prefer not to say” was coded as a separate category and not mapped to NA. The variable is set to “True” if the supervisor was selected and to “False” if the supervisor was not selected.

Perpetrator of experienced harassment coworker	harassment_experienced_perp_coworker	0.0	… if a coworker was selected.

Perpetrator of experienced harassment administrative personnel	harassment_experienced_perp_administration	0.0	… if administrative personnel was selected.

Perpetrator of experienced harassment student	harassment_experienced_perp_student	0.0	… if a student was selected.

Perpetrator of experienced harassment prefer not to say	harassment_experienced_perp_pns	0.0	… if “I prefer not to say” was selected.

Reason of experienced harassment gender	harassment_experienced_reason_gender	0.0	Derived from a question about the reason for the experienced harassment that allowed for selecting more than one answer option. The variable is set to “True” if gender was selected and to “False” if gender was not selected.

Reason of experienced harassment sexual orientation	harassment_experienced_reason_sexorient	0.0	… if sexual orientation was selected.

Reason of experienced harassment religion	harassment_experienced_reason_religion	0.0	… if religion was selected.

Reason of experienced harassment ethnicity	harassment_experienced_reason_ethnicity	0.0	… if ethnicity was selected.

Reason of experienced harassment culture	harassment_experienced_reason_culture	0.0	… if culture was selected.

Reason of experienced harassment hierarchy	harassment_experienced_reason_hierarchiy	0.0	… if hierarchy was selected.

Reason of experienced harassment age	harassment_experienced_reason_age	0.0	… if age was selected.

Reason of experienced harassment disability	harassment_experienced_reason_disability	0.0	… if disability was selected.

Reason of experienced harassment other	harassment_experienced_reason_other	97.5	The variable contains the anonymised free-text answers that respondents were asked to provide if “other” was selected.

Reason of experienced harassment don’t know	harassment_experienced_reason_idk	0.0	… if “I don’t know” was selected.

Reason of experienced harassment prefer not to say	harassment_experienced_reason_pns	0.0	… if “I prefer not to say” was selected.


Note: Variable content, variable label in the data set, number of missing responses – e.g. where respondents chose “I prefer not to say” or chose to terminate the survey before reaching the given question –, and processing steps for the CUF data file for variables in the “Working- and studying environment – harassment” subsection of the survey.

**Table 7:** Variables in the “Leadership and supervision” section of the survey.


VARIABLE CONTENT	VARIABLE LABEL	% MISSING	RESPONSE SCALE	RESPONSE SCALE NUMERIC

Leader demands item 1	leader_demands_1	33.1	Fully disagree, Somewhat disagree, Neither disagree/nor agree, Somewhat agree, Fully Agree, Does not apply	1, 2, 3, 4, 5, NA

Leader demands item 2	leader_demands_2	33.3	see above	1, 2, 3, 4, 5, NA

Leader demands item 3	leader_demands_3	33.1	see above	1, 2, 3, 4, 5, NA

Leader demands item 4	leader_demands_4	33.5	see above	1, 2, 3, 4, 5, NA

Leader demands item 5	leader_demands_5	33.5	see above	1, 2, 3, 4, 5, NA

Leader demands item 6	leader_demands_6	33.3	see above	1, 2, 3, 4, 5, NA

Leader lack of care item 1	leader_lack_of_care_1	30.7	Fully disagree, Somewhat disagree, Neither disagree/nor agree, Somewhat agree, Fully Agree, Does not apply	1, 2, 3, 4, 5, NA

Leader lack of care item 2	leader_lack_of_care_2	30.6	see above	1, 2, 3, 4, 5, NA

Leader lack of care item 3	leader_lack_of_care_3	31.6	see above	1, 2, 3, 4, 5, NA

Leader lack of care item 4	leader_lack_of_care_4	30.8	see above	1, 2, 3, 4, 5, NA

Supervisor exists	supervisor_exists	27.5	Yes, No	True, False

Supervisor integrity item 1	supervisor_integrity_1	50.3	Fully disagree, Somewhat disagree, Neither disagree/nor agree, Somewhat agree, Fully Agree	1, 2, 3, 4, 5

Supervisor integrity item 2	supervisor_integrity_2	50.0	see above	1, 2, 3, 4, 5

Supervisor integrity item 3	supervisor_integrity_3	50.2	see above	1, 2, 3, 4, 5

Supervisor integrity item 4	supervisor_integrity_4	50.8	see above	1, 2, 3, 4, 5

Number of meetings with supervisor	n_meetings_supervisor	51.8	Hardly ever/Never, Seldom, Sometimes, Often, Extremely often/Always	no mapping


Note: Variable content, variable label in the data set, number of missing responses – e.g. where respondents chose “I prefer not to say” or “Does not apply” (e.g., leader demands and leader lack of care scales), chose to terminate the survey before reaching the given question, or were not shown a question because of a filter (e.g., supervisor integrity scale and number of meetings with supervisor) –, answer options on scales, and numerical mapping of the answer options in the data files for variables in the “Leadership and supervision” section of the survey.

### 2.2 Time of data collection

Data was collected from September 15, 2023 to August 26, 2024.

### 2.3 Location of data collection

The survey targeted researchers working in universities and non-university research organisations in European countries. However, since the survey was distributed via an open registration link, some researchers from non-European countries were also reached by the survey and we did not exclude these researchers from the dataset. Table 1 lists the number of responses per country for countries with more than 24 responses. In total, 84.6% of responses came from researchers that work in a European country, 3.1% from Asia, 0.9% from Africa, and 11.5% of respondents did not disclose the country in which they work or the country was censored due to a low number of responses from that country.

### 2.4 Sampling, sample and data collection

The survey was advertised by members of the ReMO consortium via mailing lists of institutions and worker’s representations, social media channels, and word-of-mouth sharing of the registration link between colleagues and friends. Participation was voluntary and participants were not compensated. From all respondents, 61.2% report that the survey reached them via an institutional mailing list, 19.1% via colleagues, 10.7% via social media, 6.3% via worker’s representations, and 2.8% via friends.

Researchers worldwide could register for the survey with their email address. Personalized links were used to avoid duplicated responses, to enable individuals to return to the survey at a later point if they were unable to finish it in one session, to enable researchers to track individuals across survey waves using a unique anonymized identifier, and to enable individuals to request deletion of their data (see also Section “Data anonymisation and ethical issues” below for details). However, dissemination efforts focused on European countries. The survey targeted researchers of the following career stages as defined by the EURAXESS career stage taxonomy:^3^ R1 (first stage researcher), R2 (recognised researcher), R3 (established researcher), and R4 (leading researcher). Students and administrative or technical personnel were not included. In total, 4,296 researchers responded and 3,101 (72.2%) of responses were complete. As shown in Table 1, the percentage of researchers working in a given country that responded to the survey varied widely, from 0.01% of researchers working in Great Britain to 19.74% of researchers working in Montenegro. Among responses from European countries, researchers from eastern European countries, particularly Albania, North Macedonia and Montenegro, are overrepresented whereas researchers from Great Britain, Germany and the Netherlands are underrepresented.

**Figure 1:**
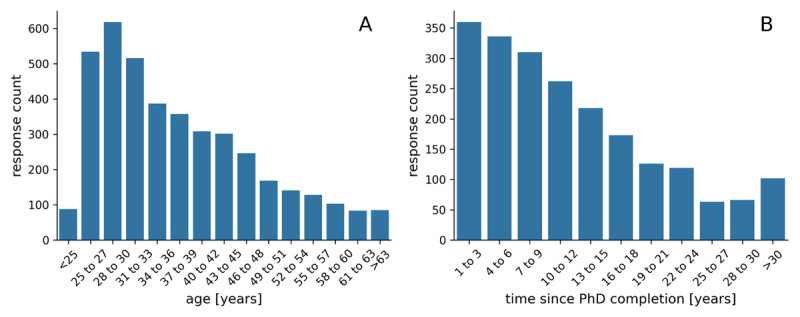
Distribution of respondent age (panel **A**) and time since PhD completion (panel **B**).

Of all respondents who disclosed their gender (93.1%), 63.7% reported to be female. As shown in Table 1, responses from female researchers are over-represented in the sample when compared to the population of female researchers in the respective countries. While an analysis of over- and underrepresentation with respect to career stage and field of research would be very advantageous to contextualize our results, we cannot provide such an analysis due to a lack of comprehensive data on the number of researchers for different career levels and research fields in different countries. The average age of respondents is 38.1 years with a standard deviation of 10.7 years. The distribution of respondent age binned into brackets of 3 years is shown in Figure 1A.

Of all respondents who disclosed their career level, 45.1% reported to be a first stage researcher (EURAXESS career level R1), 21.3% to be a recognized researcher (R2), 22.5% to be an established researcher (R3), and 10.4% to be a leading researcher (R4). An additional 1.5% report to be a researcher without a PhD degree and without the intention to pursue a PhD degree.

Out of all respondents, 2,134 (46.5%) report that they had completed a PhD degree by the time of taking the survey. Of these respondents, the mean time since completion of the PhD degree is 12.2 years with a standard deviation of 9.1 years. The distribution of time since PhD completion for respondents with a PhD degree binned into time ranges of three years is shown in Figure 1B.

Of all respondents who disclosed their research field (93.6%), 33.6% reported to work in social and human sciences, 30.4% in life sciences, 10.0% in engineering and information science, 8.1% in environment- or geoscience, 5.6% in chemistry, 5.2% in physics, 4.7% in economics, and 2.3% in mathematics.

### 2.5 Materials/Survey instruments

The survey was composed of five sections, dedicated to the collection of (i) demographic information, (ii) mental health outcomes, (iii) working and studying conditions, (iv) the working environment, and (v) leadership and supervision. We describe the content of each of these sections in detail below. The questionnaire was implemented in LimeSurvey in six different languages (English, Spanish, French, Italian, German, and Hebrew). Initially, only implementation of the questionnaire in the first five languages was planned, as they provided a good balance between coverage of large portions of the European population and resources and expertise available for translation in the consortium. However, since a Hebrew-speaking translation team volunteered to provide a Hebrew translation, we added Hebrew as a sixth language. We used validated translations of the standardized questionnaires wherever possible. If no translation existed, translation was carried out and validated by members of the ReMO consortium following the Translation, Adjudication, Pretest, and Documentation (TRAPD) approach (Walde & Völlm, 2023) – see also section “Quality Control” below for details. In total, 3,271 (71.2%) participants took the survey in English, 536 (11.7%) in French, 236 (5.1%) in Spanish, 144 in German (3.1%), 106 (2.3%) in Italian, and 4 (0.1%) in Hebrew. The LimeSurvey questionnaire implementation including all translations is available for download alongside the data set.^4^

#### 2.5.1 Demographics

First, respondents were asked to provide the career level of their primary position, following the EURAXESS researcher career stage taxonomy that classifies researchers into four career stages. In addition, the question provided the option to answer in a free-text field. Respondents who indicated that they did not hold a research position were excluded from the survey at this point. A substantial number of respondents (1.5%) answered that they were researchers but did not have a PhD degree and had no intention of pursuing a PhD degree. To account for these respondents, an additional category “researcher without a PhD degree and not pursuing a PhD degree” was introduced and the corresponding free-text answers were mapped to this category.

Respondents were then asked to provide information about their gender, year of birth, nationality, country of their main place of work, research field- and subfield, civil status, information about whether their partner pursued paid work (if not single), number of children in the household, satisfaction with their income, and year of completion of their PhD degree (for career levels R2, R3, and R4).

Information about the content of each variable contained in the demographics section of the survey, the name of the variable in the data files, the percentage of missing responses, and a short summary of the processing steps applied to each variable in the Campus Use file (CUF) is provided in Table 2.

In the second section of the survey, we asked respondents about mental health outcomes (e.g., burnout, depression, stress, anxiety, and well-being), as well as work-engagement as a primary work-related outcome, and resilience as a personal resource and an important moderator. To measure each of these constructs we used standardized and validated psychometric scales. We preferred scales that were free to use for research purposes and for which validated translations existed in as many of the survey languages as possible. Below, we briefly describe the scales used, the number of items they contain, their English language sources and the sources of the translations. If no translation is explicitly referenced, translation was carried out by members of the ReMO consortium. Information about the number of items in each scale contained in the mental health outcomes section of the survey, the name of the associated variables in the data files, the percentage of missing responses, and the response scale – both the rating scale used in the measure and its numerical mapping following the mapping suggested by the developers of the scale – are provided in Table 3.

**Burnout:** Burnout was measured using the 12-item Burnout Assessment Tool (BAT) developed by Schaufeli et al. (2020), with answer options on a five-point scale ranging from “never” to “always”. The instrument contains four subscales relating to exhaustion (items 1, 2, and 3), mental distance (items 4, 5, and 6), cognitive impairment (items 7, 8, and 9), and emotional impairment (items 10, 11, and 12). The authors of the BAT also provide translations for all other survey languages except Hebrew.

#### 2.5.2 Mental health outcomes

**Depression, stress and anxiety:** Depression, stress, and anxiety were measured using the 21-item Depression Anxiety Stress scale (DASS21) developed by Lovibond and Lovibond (1995), with answer options on a four-point scale ranging from “did not apply to me at all” to “applied to me very much or most of the time”. Depression is measured by averaging items 3, 5, 10, 13, 16, 17, and 21 of the scale. Anxiety is measured by averaging items 2, 4, 7, 9, 15, 19, and 20 of the scale. Stress is measured by averaging items 1, 6, 8, 11, 12, 14, and 18 of the scale. We used the Spanish translation of the DASS 21 by Daza et al. (2002), the French translation by Martin (2012), the Italian translation by Bottesi et al. (2015), the German translation by Nilges and Essau (2015), and the Hebrew translation by Puls (2016).

**Well-being:** Well-being was measured using the 5-item scale developed by the World Health Organization – The World Health Organization-Five well-being index (WHO-5) (Staehr, 1998), with answer options on a 6-point scale ranging from “all of the time” to “at no time”. The World Health Organization provides translations of the scale in all survey languages.

**Work-engagement:** Work-engagement was measured using a brief three-item scale (UWES) developed by Schaufeli et al. (2017), with answer options on a six-point scale ranging from “never” to “always”. The authors of the scale also provide translations for all other survey languages except Hebrew.

**Resilience:** Resilience was measured using the 6-item Brief Resilience Scale (BRS) developed by Smith et al. (2008) with answer options on a five-point scale ranging from “strongly disagree” to “strongly agree”. We used the Spanish translation by Rodríguez-Rey et al. (2016), the French translation by Jacobs and Horsch (2019), the Italian translation by Recipes for Well-being (2025), and the German translation by Chmitorz et al. (2018).

#### 2.5.3 Working- and studying conditions

In the third section of the survey, we asked respondents about their working- and studying conditions using a number of items constructed for purpose. We explicitly included “studying” conditions alongside “working” conditions in the wording of the questions after receiving feedback during testing from doctoral researchers who perceive themselves as students and do not relate to questions about “working” conditions. We describe each question and reasoning behind including it in the survey below. Information about the content of each variable contained in the working- and studying conditions section of the survey, the name of the variable in the data files, the percentage of missing responses and a short summary of the processing steps applied to each variable in the Campus Use file (CUF) are provided in Table 4.

**Main and secondary place of work:** First, we asked respondents about their main place of work. Recording this information is crucial, since according to the Individual, Group, Leader, Organisation, and the Outside Context (IGLOO) model (Nielsen et al., 2018) it is an important source of variance for mental health outcomes. For participants from European countries, we provided a drop-down menu with a closed list of research institutions for each country (Research Organization Registry, 2023). For respondents from non-European countries we only provided a free-text field where participants could enter the name of their institution. This choice was made due to practical reasons: providing a drop-down menu with research institutions for each country of the world would have meant including an excessive number of institutions. During testing we found out that this repeatedly caused the LimeSurvey questionnaire to become unresponsive or crash. Reducing the number of institutions included in the drop-down menus resolved the problem. Due to our geographical focus on European countries, we decided to include only closed lists of institutions there, and provide respondents from other parts of the world with a free-text field to name their institution. We also asked respondents if they had other workplaces in addition to their main place of work, after receiving feedback during survey testing that this is common for many researchers in European countries.

**Contract situation and overwork:** Temporary working contracts and the associated precarious working conditions in academia have been discussed as a primary reason for deteriorating mental health in researchers (e.g. Chia et al., 2024). Therefore, we assessed the contract situation of respondents by asking them about the duration of their current working contract, whether it is a tenure-track position (e.g., has the option of becoming a permanent position after fulfilling some specified criteria), or is tenured. Similarly, long working hours have been associated with unfavourable mental health outcomes (e.g., Sang et al., 2015). Therefore we asked participants to provide information about the number of working hours specified in their working contract, and the number of hours they actually work per week.

**Time spent on different tasks and professional development:** To gain a better understanding of the characteristics of the work performed by researchers and their relation to mental health outcomes, we asked respondents to provide information about the percentage of working time they regularly spend on research, teaching, applying for research funding, mentoring, administrative tasks, and other tasks. The different percentages provided for each task were required to sum to 100%. In addition, we asked respondents to provide information about the number of days they spent on professional development (e.g., attending conferences, courses, workshops, and training) in the last 12 months.

**Research outputs:** While we recognize the limited informative value of such an indicator, the number of publications is still oftentimes used by managers of research institutions to assess the performance of researchers in practice, and by research funders to assess the performance of institutions. We therefore considered it an important secondary outcome that could potentially capture the relationship between mental health outcomes, working conditions, and research outputs. However, in different research fields there are very different types of publications that are considered of value. We therefore asked respondents to provide information about the number of research outputs they published over the last 12 months in five different categories: peer reviewed research papers, peer reviewed conference articles, monographs, patents and “other”. Furthermore, different research fields also have vastly different rates of publishing. We therefore caution against the use of the reported research outputs for comparisons between different disciplines.

**Intentions of leaving academia and recommending academia:** Intentions of leaving academia or the willingness to recommend academia as a career are often considered important outcomes for human resource managers in institutions. We therefore assessed these two dimensions by asking respondents how often they seriously considered leaving academia in the last 12 months, and whether they would generally recommend a career in research and higher education to early-career researchers today. Both questions allowed for answers on a five-point scale ranging from “hardly ever/almost never” to “extremely often/always” for intentions of leaving academia, and from “fully disagree” to “fully agree” for the willingness to recommend working in academia.

#### 2.5.4 Working and studying environment

Next to the more qualitative indicators characterising the working and studying conditions described in the section above, we relied on a number of validated scales to assess the working and studying environment. Again, we preferred scales that were free to use for research purposes and for which validated translations existed in as many of the survey languages as possible. Below, we briefly describe the scales used, the number of items they contain, their English language sources, and the sources of the translations to the other survey languages. Similar to the scales described in the “Mental health outcomes” section, if no translation is explicitly referenced, translation was carried out and validated by members of the ReMO consortium (see also section “Quality Control” below). Information about the number of items in each scale contained in the working and studying environment section, the name of the associated variables in the data files, the percentage of missing responses, and the response scale – both the scale used in the questionnaire and its numerical mapping following the mapping suggested by the developers of the scale– are provided in Table 5.

**Predictability, role clarity, sense of community, emotional support, and instrumental support:** To assess the working and studying environment in terms of its predictability, role clarity, the prevalent sense of community, as well as the emotional and instrumental support available to the respondent, we used scales contained in the Copenhagen Psychosocial Questionnaire (COPSOQ) developed by Burr et al. (2019). We used the Spanish translation by Moncada et al. (2021), and the French, Italian and German translations by Peter et al. (2022). Within COPSOQ, predictability is assessed with three items, role clarity with four items, and sense of community with three items. We adapted the COPSOQ questions for emotional and instrumental support to the academic working and studying environment, asking respondents about how often their direct supervisor, superior (e.g., department head, etc.), colleagues, administrative personnel, or partner, friends/family members or other people in their personal community are willing to listen to their problems at work (emotional support, five items) or provide help and support (instrumental support, five items). Each scale had answer options on a five-point scale ranging from “hardly ever/almost never” to “extremely often/always” for sense of community, instrumental support, and emotional support, and from “to a very large extent” to “to a very little extent” for predictability and role clarity.

**Policy perceptions:** We assessed the respondent’s perceptions of workplace policies for safety, health and well-being using the 7-item scale developed by Sorensen et al. (2018), with answer options on a five-point scale ranging from “to a very large extent” to “to a very little extent”.

**Job insecurity:** We assessed the respondent’s feeling of job insecurity using the 4-item scale developed by Vander Elst et al. (2014), who also provide a Spanish translation. The scale has answer options on a five-point scale, ranging from “fully disagree” to “fully agree”.

**Job control:** The feeling of control over the day-to-day work of respondents was assessed using the 3-item scale developed by Hackman and Oldham (1975), and the German translation by Ehrlich (2008). The scale has answer options on a five-point scale ranging from “fully disagree” to “fully agree”.

**Interpersonal conflict:** To assess the extent of interpersonal conflict present at the workplace, we used the 3-item scale developed by Færgestad (2017), with answer options on a five-point scale ranging from “fully disagree” to “fully agree”.

**Work-family conflict:** To assess how compatible work/studying is with care obligations of respondents, we used the 5-item scale developed by Netemeyer et al. (1996), translated to Spanish by Moncada et al. (2021), to Italian by Colombo and Ghislieri (2008), and to German by Nübling et al. (2005). The scale has answer options on a five-point scale ranging from “fully disagree” to “fully agree”.

**Illegitimate tasks:** We assessed the prevalence of tasks the respondents have to do at work that they feel are illegitimate, using the scale developed by Semmer et al. (2010) with a translation to Spanish by Valdivieso et al. (2021), and to German by Jakobshagen (2006). The scale has four items and answer options on a five-point scale ranging from “hardly ever/almost never” to “extremely often/always”.

**Work-related stress:** In addition to general stress which we assessed using DASS 21 (as described in the “Mental health outcomes” section), we also assessed specific work-related stress using a three-item scale developed by Edwards et al. (2008), with answer options on a five-point scale ranging from “hardly ever/almost never” to “extremely often/always”.

**Job satisfaction:** Finally, we also assessed overall job satisfaction asking respondents “All things considered, how satisfied are you with your job?”, providing a reply scale between 0 “very dissatisfied” to 10 “very satisfied”.

#### 2.5.5 Perceived and experienced harassment

Given recent attention directed at instances of power abuse, bullying and harassment at academic institutions (see e.g., Roupas, 2025), we dedicated a relatively large part of the “Working and studying environment” section to assess the respondent’s perceptions of and experiences with harassment, as well as the perpetrators of the harassment and the perceived reason for the harassment. To this end, to assess perceived harassment we asked respondents “Have you noticed co-workers being subjected to degrading experiences or harassment at your place of work or study in the last 6 months?”. To assess experienced harassment, we asked respondents “Have you been subjected to degrading experiences or harassment at your place of work or study in the last 6 months?”.

For both perceived and experienced harassment, if a respondent answered “yes” to the question, we followed up with the question “Who was the perpetrator or the perceived/experienced degrading experiences or harassment?” which provided the non-exclusive response options “supervisor”, “coworker”, “I prefer not to say” and a free-text response field. Analysis of the free-text answers to this question yielded a high number of answers indicating students and administrative personnel as perpetrators in perceived and experienced harassment, warranting the creation of these categories which were coded from the respective free-text answers.

We also asked respondents to state the basis for the perceived/experienced harassment, providing the non-exclusive response options “gender”, “sexual orientation”, “religion”, “ethnicity”, “culture”, “hierarchy”, “age”, “disability”, “I don’t know”, “I prefer not to say” and a free-text response field.

Information about the content of each variable contained in the harassment subsection of the working environment section, the name of the variable in the data files, the percentage of missing responses and a short summary of the processing steps applied to each variable are provided in Table 6.

#### 2.5.6 Leadership and supervision

The Individual, Group, Leader, Organisation, and the Outside Context (IGLOO) model (Nielsen et al., 2018) identifies the relationship with the respective superiors in the workplace as an important determinant of work-related mental health outcomes. In the academic context, we differentiate between “leaders” and “supervisors” and explain this differentiation to participants in an explainer text provided at the start of the survey section. Here, “leader” refers to that person who directly influences how work in a team is organized. This could for example be a group leader or department head, depending on the researcher’s position. “Supervisor” refers to a person that has a say in how research or studies are conducted. We mainly expected doctoral researchers and potentially PostDocs to have supervisors, whereas we expected all researchers to have leaders – even though in the case of leading researchers (e.g., professors) there is oftentimes no superior that exerts close influence on how work is conducted. For this reason, we also provided a “does not apply” answer option to questions in this section.

We assessed the characteristics of the leader using two validated scales and the characteristics of the supervisor with one scale, described below. Information about the number of items in each scale contained in the leadership and supervision section of the survey, the name of the associated variables in the data files, the percentage of missing responses, and the response scale – both the response scale used in the questionnaire and its numerical mapping following the mapping suggested by the developers of the scale – are provided in Table 7.

**Leader demands & leader lack of care:** Almeida et al. (2022) provide a 6-item scale to assess the characteristics of demands a leader has on their team, and a 4-item scale assessing the (lack of) care of the leader. Both scales have answer options on a five-point scale ranging from “fully disagree” to “fully agree” and include an additional “does not apply” item for respondents that do not feel that they have a leader they report to in their working environment.

**Supervisor integrity:** We first ask respondents whether they currently have a person they consider their supervisor. If this is the case, we use the 4-item scale developed by Colquitt and Rodell (2011) to assess the respondent’s perception of the integrity of their supervisor. The scale provides answer options on a five-point scale, ranging from “fully disagree” to “fully agree”. Lastly, we also ask respondents to provide information about how often they talk with their supervisor about how well they carry out their work.

### 2.6 Quality Control

Before deploying the survey, the questionnaire was subjected to a number of testing and piloting phases: We started with the creation of a long version of the questionnaire in English, which was created in tandem with the development of the study aim and based on theoretical concepts describing the relationship of mental health and workplace characteristics on different IGLOO-levels. This questionnaire was then piloted with 10 testers that were all members of the ReMO consortium, with the aim of gauging the time necessary to complete the survey, targeting a completion time of not more than 15 minutes. After this initial round of testing, individual items and item batteries were excluded to shorten completion time with a priority on the removal of items that were not free to use for research purposes or where testers felt that items had a large overlap with other items or scales contained in the survey.

Next, the shortened version of the English questionnaire was tested with the aim of checking for question clarity with a specific focus on capturing the varying national contexts in which researchers work in Europe. Testing was undertaken by 17 members of the ReMO consortium that were selected to cover a large number of European countries. After this round of testing, a number of questions regarding the work context of researchers were rephrased and a small number of questions was added. Completion time of the survey in this testing round was approximately 15 minutes among the testers.

Lastly, after finalising the English language version, the questionnaire was translated to French, Spanish, German, Italian and Hebrew, using validated translations of the instruments where possible. Where no validated translations existed or items did not originate from validated scales in the first place, questions were translated following the Translation, Review, Adjudication, Pretest, and Documentation (TRAPD) approach (Walde & Völlm, 2023). Specifically, a native speaker of the target language translated the questionnaire and the translation was quality checked by a second native speaker of the target language. Differences between the two translators were discussed and resolved. All questionnaire versions were tested again by at least one native speaker to identify errors in the translations or in the technical implementation of the questions in LimeSurvey. Changes to item formulations were documented. No changes to the content of the survey were made at this point.

After collection of the data, responses from individuals that completed the survey in multiple sittings were linked, and responses from individuals who indicated that they do not hold an academic position (in total 294) were removed, leaving 4,296 responses. No further quality control measures such as attention checks or measurement invariance tests across countries were applied during or after data collection.

### 2.7 Data anonymisation and ethical issues

The study design and data collection plan was evaluated by the Ethics Commission of the Leibniz University Hannover and approved under ethics vote EV LUH 04/2023.

The invitation to the survey was distributed via mailing lists and social media. It contained a link that led prospective participants to a landing page where they could enter their email address and consent to be contacted via email with a personalized link to participate in the survey. Email addresses that had already registered for participation in the survey were excluded from participating a second time. Before starting the survey, participants were presented with the participant information and informed consent form in English.^5^ The participant information and informed consent form were also translated into the five other survey languages and six additional languages (Croatian, Hungarian, Macedonian, Portuguese, Romanian, and Turkish) and translations were accessible via links in the English language document. Participants could only proceed with taking the survey after providing their informed consent and confirming they were 18 years or older. Participants could pause and return to the survey at any time, using the personalized access link sent to their email address.

Next to the prevention of dual participation and the ability to come back to the survey at a later point in time, being able to link participants to their email addresses is also necessary to track individuals across future survey waves. Lastly, linking respondents to their email addresses was also necessary to respect the “right to be forgotten” (Article 17 GDPR) for the personal data contained in the SUF. Given their email address, participants can request a download or deletion of their records in the SUF from the data access provider. However, since the data in the CUF is completely anonymous, no link to the original email addresses needs to be retained. Therefore, for the CUF email addresses were deleted and participants were assigned a newly generated random participant ID.

To respect the privacy of respondents, a number of anonymization steps were applied, with stricter anonymization applied to the CUF. For example, in the CUF, demographic information has been aggregated or removed to an extent that makes re-identification of individuals impossible, rendering it completely anonymous. Here, we provide a detailed description of the processing steps applied to each variable in the CUF. Processing steps applied to the SUF are described in section A2 in the Appendix. The data protection plan detailing data protection measures taken during data collection and processing was made available to respondents via a link in the participant information and informed consent form and is publicly accessible (Lasser, 2023). Due to the different levels of sensitivity of the information contained in the SUF and CUF, different access modalities apply to the two versions of the data set which are described in detail in Section 3 “Data set description and access” for the CUF and in section A3 in the Appendix for the SUF.

**Primary position:** The two categories “established researcher” (R3) and “leading researcher” (R4) were merged, and the category “researcher without a PhD degree and not pursuing a PhD degree” was set to NA due to low response counts in these categories.

**Gender:** As the number of respondents reporting a gender other than “female” and “male” was extremely low (35 in total), these entries were mapped to NA as the risk of re-identification via this variable was deemed too high.

**Age:** Participants provided their year of birth in the survey. Using this information we calculated the age of participants at the time they took the survey. Ages were aggregated into the categories <31 years, 31–42 years, and >42 years.

**Nationality:** Nationality information was removed completely as it was deemed too sensitive.

**Country of main place of work:** Only information about the continent was retained and aggregated into the categories “Europe” and “outside Europe”.

**Main place of work:** Information about the main place of work was deleted completely as it was deemed too sensitive.

**Civil status:** Information about the civil status of respondents which was originally collected in the five categories “in a relationship and cohabiting”, “in a relationship but not living together”, “married and cohabiting, “married but not living together”, and “single”, was aggregated into the two categories “in a relationship/married” and “single”.

**Partner pursues paid work:** This variable was deleted completely as it was deemed too sensitive.

**Research field:** The question about a participant’s research field originally included two levels, where the first level asked for the larger research field (e.g. “Social and Human Sciences”, “Life Sciences”, etc.), and the second level asked for the research field on a finer-grained level within the larger research field (e.g. “Psychology”, “Education and Training”, “Linguistics”, etc. for categories within the “Social and Human Sciences”). Because the risk of re-identification given any information about the finer-grained research field was deemed too high, this information was removed completely. Information about the broader research field was aggregated into the three categories “Chemistry, Physics, Mathematics”, “Engineering and Information Science; Environment or Geo-Science”, and “Social and Human Sciences; Economics”.

**Number of babies, children, adolescents, and young adults in the household:** The question about the presence of children of different ages in the household originally asked about the number of children in each of four different age categories (babies: 0–23 months, children: 2–11 years, adolescents: 12–17 years, young adults: 18–25 years). To reduce the risk of re-identification, information about the number of children was removed and only information about whether or not children in a given age category were present in the household was retained.

**Time since PhD degree:** For participants of researcher career level R2 (Recognized researcher) and above, we asked about the year in which they completed their PhD degree. Similar to the processing of participant age, we calculated the time since completion of the PhD degree and aggregated the time into the two categories 1–15 years and >15 years.

**Contract duration:** Information about the duration of the contract of a respondent was aggregated into the two categories “permanent position” and “termed contract/no contract/stipend”.

**Overwork:** The survey originally asked participants to specify both their contractually agreed upon working time, as well as the time they actually worked. Since the variable that is actually of interest for the study design is the amount of overwork, we combined information from these two questions to calculate the amount of over- or underwork in brackets of five hours, which is provided in a new variable. Answers to the two original questions were deleted from the data file.

**Time spent on research, teaching, applying for funding, mentoring, administrative tasks, and other tasks:** Information about the percentage of working time spent on different tasks was removed completely as it was deemed too sensitive.

**Research outputs:** The survey originally asked participants for the number of research outputs in five distinct categories (“peer reviewed journal article”, “peer reviewed conference article”, “monograph”, “patent” and “other”) they published in the last 12 months. We collapsed these categories, retaining only the overall number of research outputs published in the last 12 months and aggregated it into the categories 0, 1–5, and >5.

### 2.8 Existing use of data

To date, there exists no publication using the described data set. However, a number of analysis efforts led by members of the ReMO consortium are under way. In particular, work on analysing the multilevel variability of researcher mental health and testing the primary hypotheses of relationships between a number of working conditions and mental (ill) health has been pre-registered under Mol et al. (2025).

## (3) Dataset description and access

### 3.1 Repository location

The Campus Use File (CUF) is located at https://doi.org/10.21249/DZHW:remo:1.1.0.

### 3.2 Object/file name

remo_cuf_1-1-0.csv; remo_cuf_1-1-0.dta; remo_cuf_1-1-0.sav.

Please note that the version number will change in case of updates. The original files will still be available.

### 3.3 Data type

Primary data, processed data.

### 3.4 Format names and versions

Data files are provided in CSV, Stata, and SPSS file formats.

### 3.5 Language

The data is stored in British English.

### 3.6 License

Since a data usage contract is concluded (see below), a separate license is not necessary.

### 3.7 Limits to sharing

To access the data, users need to place a data access request with the service provider. To this end, after clicking on the DOI, the data set needs to be added to the “shopping cart” via the orange button in the bottom right of the page or the orange button in the left side-panel of the page. Once the data set has been added to the shopping cart, the access application needs to be completed by clicking on the shopping cart symbol in the top right of the page and then clicking “order free of charge”. To complete the process, users will be prompted to register an account with the data access provider. After registering, there are several options to select different versions of the data set (e.g., CSV, SPSS, or Stata). In addition, users will be prompted to provide minimal information about their academic position and purpose of the data access request. After accepting the General Terms and Conditions (see https://fdz.dzhw.eu/en/node/1301) the data access request can be submitted. The request will take a few working days to process since it is undergoing manual review on the side of the data access provider.

We recognise that the process of getting access to the data is relatively involved. However, with data as sensitive as mental health information, ease of data access needs to be balanced with the data protection rights of data subjects. Data access and anonymisation procedures have been balanced accordingly, in constant exchange with the project’s and the data provider’s data protection officers.

In case of questions, the data access provider offers a support service reachable under userservice@dzhw.eu.

### 3.8 Publication date

The CUF was published on April 30, 2025.

### 3.9 FAIR data/Codebook

#### Findable

The data release on the DZHW data center is assigned a globally unique and persistent identifier, e.g. DOI. The data is described with rich metadata that is associated with (i) the data package, (ii) the survey, (iii) the survey instrument, and (iv) associated publications, facilitating integration of data from future survey waves in the same reporting framework.

**Data package:** The metadata associated with the data package describes the study series (STAIRCASE survey), the responsible institution (TIB – Leibniz Information Centre for Science and Technology University Library) and funding agency (European Research Council), names and (where available) ORCIDs of project contributors, the survey design (cross-sectional), the survey data type (quantitative data), information about the person responsible for data curation (Jana Lasser), the current data release version, links to additional resources (e.g., project website and survey website), and the allowed use cases. It also contains a brief description of the data contained in the data package (e.g. the number of responses, demographic and working condition related variables recorded, and scales used). It also includes additional documents related to the data package, specifically the informed consent form and participant information in all available translations, and the data protection plan.

**Survey:** The metadata associated with the survey includes the serial number of the data collection wave, describes the researched population, sampling procedure (non-probability sample: respondent-assisted sample) and geographic coverage, the data type (quantitative data), the survey method (standardised online survey), the field period (Sep 15, 2023–Aug 26, 2024), and the net sample size (4,296).

**Survey instrument:** The metadata associated with the survey instrument includes the title, the language and the technical implementation (CAWI) of the instrument.

**Associated publications:** The associated publications will be updated continually and link to any publications originating from the published data set.

#### Accessible

The metadata is retrievable via the data set’s DOI and via other metadata hubs like DataCite. The CUF can be downloaded for teaching purposes (free of charge) after an informal usage request using the order function on the DOI landing page (see more detailed description in Section 3.7 above).

#### Interoperable

The data is provided in a number of widely used data formats, including as CSV, Stata, and SPSS data files. The metadata includes qualified references to other metadata, e.g. associated publications and (potential) future survey waves. The metadata is registered at da|ra (https://www.da-ra.de/; a DataCite registration agency) and follows the DataCite metadata standard. Further, the metadata is mapped to the DublinCore, Schema.org and DDI 3.2 metadata standards.

#### Reusable

The metadata provided alongside the data describes the data with a plurality of accurate and relevant attributes that meet domain-relevant community standards (see description of metadata under “Findable” and “Interoperable” above), including a clear and accessible data usage license and detailed provenance information.

#### Codebook

The codebook of the data set is provided as supplementary information to the article. In the codebook, we provide a description of all variables contained in the STAIRCASE data set on researcher mental health. The codebook is structured into five sections, corresponding to the sections of the online questionnaire and the subsections of section 2.5 “Materials/Survey instruments”. Each section starts with an introductory text that was shown to participants taking the survey, followed by tables that summarise the information of each variable derived from questions that were asked in the respective section. For most variables, we provide the full wording of the question text in all languages. However, due to copyright we cannot provide the wording of some items of instruments that are not published under a creative commons license. For these items, we provide the reference to the source of the question texts. However, a non-public version of this codebook containing all questions is made accessible to researchers using the Scientific Use File in the secure data processing environment.

## (4) Reuse potential

The data set from the Researcher Mental Health Observatory STAIRCASE survey holds considerable reuse potential for research and teaching given its detailed exploration of the working conditions and mental health outcomes of researchers. A key reuse potential is the facilitation of secondary analysis: While the ReMO consortium is currently engaged in a number of analysis efforts (see pre-registered multi-level analysis plan at Mol et al., 2025), there are a multitude of possible analysis avenues that remain unexplored. These include country-specific analyses for countries with a large enough number of responses, comparisons between countries, regions and research fields, and analysis foci on specific subsections of the survey, such as leadership behaviour, working conditions and experiences with harassment. Due to the use of widely established measurement instruments for mental health outcomes, such as the depression, anxiety and stress scale (DASS 21), the WHO well being measure, and the Burnout Assessment Tool (BAT), the collected data can also be combined with data from other studies that measure the same constructs to enable comparisons between different populations or across time.

The survey instrument, which has been translated to five languages next to English (Spanish, French, Italian, German, and Hebrew), is available in an open format (e.g., LimeSurvey) and will hopefully facilitate future implementations of the same or similar surveys, creating more data sets that are compatible with the first wave of data collection. Such data could help to shed light on the state of researcher mental health in regions other than Europe and the development of researcher mental health over time. This is supported by the detailed and open documentation of the data set, including a GDPR compliant data protection plan, and consent forms translated into 12 languages. Overall, we hope that the STAIRCASE survey provides a robust framework for researchers seeking to understand factors contributing to mental health issues within academic settings.

In an educational context, the Campus Use File (CUF) can serve as a valuable resource for teaching purposes, offering fully anonymized data that can be easily downloaded and incorporated into curricula. It can be used for practical training in data analysis, to exemplify the data collection process, or to discuss the ethical and data protection considerations of conducting research on sensitive topics such as mental health. The data set’s comprehensive nature ensures that it can be used to demonstrate a variety of statistical techniques, from basic descriptive analysis to more complex inferential methods. Additionally, because of its focus on a current and pressing issue, it serves as a dataset relevant to students’ future careers in research and academia, potentially heightening their engagement and the practical relevance of their learning.

One of the notable limitations of the STAIRCASE survey data is the method of recruitment of respondents via an open invite link. This could lead to a self-selection bias, where individuals experiencing higher levels of stress or those with an interest in mental health issues may have been more likely to participate. Additionally, the survey recruitment procedures varied across countries, with some dissemination teams being more thorough than others, which led to significant differences in response rates between countries. While we cannot reliably gauge the level of overrepresentation of respondents experiencing higher levels of stress or with an interest in mental health, we confirm an overrepresentation of female researchers and researchers from eastern European countries in the sample by comparing response rates to OECD data on the researcher workforce in different countries. Furthermore, the survey used only self-report measures which may be subject to individual biases and do not necessarily provide objective measures of the working conditions or mental health statuses. Lastly, the survey’s reach to respondents in non-European countries, although not excluded, is not comprehensive by any means. Therefore the global research community is severely underrepresented in the collected data. In addition, data was collected over a field phase of close to 12 months, covering two semesters and therefore two cycles of teaching, exam periods, and semester breaks. This long time period might lead to systematically different replies regarding the level of experienced stress and related mental health outcomes, depending on the time of the year the survey was taken.

In conclusion, while the STAIRCASE survey data provides rich insights into the mental health of researchers in academia, particularly within Europe, the limitations of the collected data should be taken into account when interpreting the data and when considering its broader application to policy recommendations. However, the STAIRCASE survey represents the – to our knowledge – richest data set about researcher mental health collected to date and will hopefully enable a number of follow-up studies and data collection endeavours that help to understand the connection between mental health outcomes and working conditions and change the academic system for the better.

## Additional File

The additional file for this article can be found as follows:

10.5334/jopd.136.s1 Appendix. Data from the Researcher Mental Health Observatory STAIRCASE survey.

## Footnotes

See https://w3.unece.org/SDG/en/Indicator?id=124.

See https://uis.unesco.org/sites/default/files/percentage-of-female-researchers-_in_hc_and_fte.pdf.

See https://euraxess.ec.europa.eu/career-development/researchers.

See https://metadata.fdz.dzhw.eu/de/instruments/ins-remo-ins1?page=1&size=10&type=surveys&version=1.1.0.

https://osf.io/9tr74/files/zemau.
