# Supplementary material for: Data from the Researcher Mental Health Observatory STAIRCASE Survey
Source: J Open Psychol Data. 2026 May 29;14:1. doi: 10.5334/jopd.136 (PMC13220731; doi:10.5334/jopd.136)
Supplement: Appendix. — Data from the Researcher Mental Health Observatory STAIRCASE survey. [file jopd-14-136-s1.pdf]

Data from the Researcher Mental Health Observatory STAIRCASE survey – Appendix

Authors: Jana Lasser, Stefan T. Mol, and the Researcher Mental Health Observatory Consortium

We provide the data from the survey in two versions: the Campus Use File (CUF) for teaching and testing purposes that has been fully anonymised and is accessible for download, and the Scientific Use File (SUF) to which less strict anonymisation measures apply and which is only available in a secure data processing environment. The motivation and content of the survey as well as the data processing steps applied to the CUF are described in the main manuscript. Here, we describe the data processing steps, data protection measures, and access modalities specific to the Scientific Use File (SUF).

A1 Data processing steps of the Scientific Use File

A1.1 Demographics

Information about the content of each variable contained in the demographics section of the survey, the name of the variable in the data files and the percentage of missing responses are provided in Table 2 in the main manuscript. A short summary of the processing steps applied to each variable in the SUF is provided in Table A1 below.

Table A1: Variables in the "Demographics" section of the survey (SUF version).

variable content	variable label	SUF processing
Participant ID	participant_id	Created by applying a salted hash function to the participant's email address.
Primary position	primary_position	
Gender	gender	"non binary" set to NA
Age	age	Calculated age from year of birth, binned into 3-year brackets with bottom and top coding, e.g. <25 and >63 years.
Nationality	nationality	Retained only region for countries with <27 responses.
Nationality region	nationality_region	Derived from nationality using a mapping of countries to regions provided by the UN (see www.unstats.un.org/unsd/methodology/m49/#ftn13). For regions with <28 responses only the continent was retained.
Nationality continent	nationality_continent	Derived from nationality_region using UN region mapping (see above). Continents with < 100 responses were set to NA.
Place of work country	country_of_work	Retained only region for countries with <24 responses.
Place of work region	country_of_work_region	Derived from country_of_work using UN region coding. Retained only continent for regions with <26 responses.
Place of work continent	country_of_work_continent	Derived from country_of_work_region using UN region coding. Continents with <100 responses were set to NA.
Research field	research_field	Mapped free-text answers to existing categories.
Research field - subfield	NA	Deleted
Civil status	civil_status	Mapped free-text answers to existing categories.
Partner work	partner_work	
Babies in household	babies_in_household	Derived from a question asking for the number of babies by mapping 0 to "no" and numbers >0 to "yes".
Children in household	children_in_household	Derived from a question asking for the number of children by mapping 0 to "no" and numbers >0 to "yes".

Adolescents in household	adolescents_in_household	Derived from a question asking for the number of adolescents by mapping 0 to “no” and numbers >0 to “yes”.
Young adults in household	young_adults_in_household	Derived from a question asking for the number of young adults by mapping 0 to “no” and numbers >0 to “yes”.
Income	income	
Time since PhD degree	years_since_phd_completion	Calculated age from year of PhD completion, binned into 3-year brackets with top coding, e.g. >30 years.

Note: Variable content, variable label in the data set, and processing steps for the SUF data file for variables in the “Demographics” section of the survey.

A1.2 Working- and studying conditions

Information about the content of each variable contained in the working- and studying conditions section of the survey, the name of the variable in the data files and the percentage of missing responses are provided in Table 4 in the main manuscript. A short summary of the processing steps applied to each variable in the SUF is provided in Table A2 below.

Table A2: Variables in the “Working- and studying conditions” section of the survey (SUF version).

**variable content**	**variable label**	**SUF processing**
Main place of work	main_place_of_work_hashed	Institution names were hashed to reduce the risk of re-identification.
Additional places of work	other_work	
Contract duration	contract_duration	The original question provided the answer option “I have a tenure-track position” that was non-exclusive with answer options “Within the next 12 months”, “In 1-2 years” “In 2-3 years”, and “In more than 3 years”. This answer was transformed into a separate column.
Tenure track	tenure_track	Created by mapping the response “I have a tenure track position” from the contract duration question (see above) to “yes” and all other responses to “no”.
Overwork	working_hours_overtime	Created by subtracting the answer to the question “How many working hours per week are specified in your contract or funding for your main place of work?” from the answer to the question “How many hours per week do you actually work (on average)?”
Time spent on teaching	percent_teaching	The question did not include an “I prefer not to say” answer option. If respondents did not provide a number, the entry was mapped to NA.
Time spent on research	percent_research	The question did not include an “I prefer not to say” answer option. If respondents did not provide a number, the entry was mapped to NA.
Time spent on applying for research funding	percent_funding	The question did not include an “I prefer not to say” answer option. If respondents did not provide a number, the entry was mapped to NA.
Time spent on mentoring	percent_mentoring	The question did not include an “I prefer not to say” answer option. If respondents did not provide a number, the entry was mapped to NA.
Time spent on administrative tasks	percent_admin	The question did not include an “I prefer not to say” answer option. If respondents did not provide a number, the entry was mapped to NA.
Time spent on other tasks	percent_other	The question did not include an “I prefer not to say” answer option. If respondents did not provide a number, the entry was mapped to NA.
Research output	research_output	The original question asked for the number of research outputs in the categories “Peer reviewed journal article”, “Peer reviewed conference article”, “Monograph”, “Patent” and “Other”. The variable was created by adding the numbers provided for each of the five output subcategories. Not providing a number defaulted to 0, there was no “I prefer not to say” answer option.
Professional development	days_professional_development	
Leaving academia	leaving_academia	
Recommending academia	recommend_academia	

Note: Variable content, variable label in the data set, and processing steps for the SUF data file for variables in the “Working- and studying conditions” section of the survey.

A1.3 Perceived and experienced harassment
The processing steps applied to each variable in the SUF are the same as for the CUF and provided in Table 6.

A2 Data protection measures of the Scientific Use File
To preserve the de-facto anonymity of participants in the SUF, email addresses were converted into participant IDs using a salted hash function. The salt of the hash function serves as the secret, without which linking of the participant ID and the email address is not possible. The secret is deposited with the data protection officer of the data storage provider and protected by organisational and legal data protection measures. It can only be accessed for the purpose of identifying the records of individuals to download or delete their data under GDPR, given their email address.

Next to removing any direct identifiers (e.g., email addresses) from the data, a number of processing steps were applied to the demographic information and information about the working and studying conditions provided by the participants to reduce the risk of re-identification. We describe these steps for the affected variables below.

Gender: As the number of respondents reporting a gender other than "female" and "male" was extremely low (35 in total), these entries were mapped to NA as the risk of re-identification via this variable was deemed too high.

Age: Participants provided their year of birth in the survey. Using this information we calculated the age of participants at the time they took the survey. Ages were aggregated into bins of three years each, applying bottom and top cutoffs at <25 and >63 years as the number of respondents below and above these ages was exceedingly small.

Nationality: For respondent nationality, we only retained country information for countries with more than 27 responses. All other country records were mapped to NA and only information about the geographic region (e.g. "western europe", "south-eastern asia" etc.) was retained. To map countries to regions we used the mapping provided by the UN[1]. We followed the same process for regions, retaining only information for regions with more than 28 responses and mapping the other regions to continents, again using the mapping of regions to continents provided by the UN. Lastly, we only retained information for continents with more than 100 responses.

Country of main place of work: The same process as described for nationality above was applied to information about the country where the respondent's main place of work was located. Here, the cutoffs were 24 or more responses for retaining country information, 26 or more responses for retaining region information, and 100 or more responses for retaining continent information.

[1] See http://unstats.un.org/unsd/methodology/m49/#ftn13.

Main place of work: We did not further process information provided in free-text fields, since it would have required too much time to extract and unify institution names. As a result, the country of the main place of work for respondents that provided a free-text answer was set to NA. For the information provided via the drop-down menus, we replaced institution names with institution IDs by applying a salted hash function to the institution names. This allows for the identification of individuals who work at the same institution – which is an important feature to enable the multilevel statistical analysis following the IGLOO model (Nielsen et al., 2017) – without revealing the name of the institution, which would have greatly increased the risk of re-identification of individuals.

Research field: The question about a participant's research field originally included two levels, where the first level asked for the larger research field (e.g. "Social and Human Sciences", "Life Sciences" etc.), and the second level asked for the research field on a finer-grained level within the larger research field (e.g. "Psychology", "Education and Training", "Linguistics", etc. for categories within the "Social and Human Sciences"). Because the risk of re-identification given any information about the finer-grained research field was deemed too high, this information was removed completely.

Number of babies, children, adolescents, and young adults in the household: The question about the presence of children of different ages in the household originally asked about the number of children in each of four different age categories (babies: 0-23 months, children: 2-11 years, adolescents: 12-17 years, young adults: 18-25 years). To reduce the risk of re-identification, information about the number of children was removed and only information about whether or not children in a given age category were present in the household was retained.

Time since PhD degree: For participants of researcher career level R2 (Recognized researcher) and above, we asked about the year in which they completed their PhD degree. Similar to the processing of participant age, we calculated the time since completion of the PhD degree and then mapped the information to time brackets of three years each, applying a cutoff at >30 years.

Overwork: The survey originally asked participants to specify both their contractually agreed upon working time, as well as the time they actually worked. Since the variable that is actually of interest for the study design is the amount of overwork, we combined information from these two questions to calculate the amount of over- or underwork in brackets of five hours, which is provided in a new variable. Answers to the two original questions were deleted from the data file.

Research outputs: The survey originally asked participants for the number of research outputs in five distinct categories ("peer reviewed journal article", "peer reviewed conference article", "monograph", "patent" and "other") they published in the last 12 months. We collapsed these categories, retaining only the overall number of research outputs published in the last 12 months.

After applying these processing steps, the SUF contains a de facto anonymous data set, as any potential re-identification would require a disproportionately high level of effort, if possible at all (considering also that the data access mechanism described below prevents the integration or injection of external data.). In addition to the aforementioned anonymisation measures and the controlled data access, contractual regulations with the data users prohibit the de-anonymisation of the SUF.

A3 Data access modalities of the Scientific Use File

The SUF can be accessed remotely in a secure data analysis environment via a virtual desktop after signing a Data Use Agreement (see sections "Limits to sharing" below).

A3.1 Repository location

Access to the Scientific Use File (SUF) can be requested via https://doi.org/10.21249/DZHW:remo:1.1.0 from May 1, 2026.

A3.2 Object/file name

remo_suf_1-1-0.csv; remo_suf_1-1-0.dta; remo_suf_1-1-0.sav

Please note that the version number will change in case of updates. The original files will still be available.

A3.3. Limits to sharing

As the SUF contains potentially identifying information, access to it is subject to gatekeeping which ensures the technical, organisational and legal measures necessary for adequate data protection are taken. Upon signing a Data Use Agreement, the data storage provider (DZHW) will supply remote access to a secure analysis environment in which the SUF can be accessed. Remote access will allow users to establish an encrypted connection to a DZHW server that holds the SUF and pre-installed analysis programs such as R, Python and SPSS Permanent access to commercial, fee-based software such as SPSS cannot be ensured. Free and open-source analysis tools may be installed where technically feasible. Users will then be able to perform the desired analysis on this server, including the upload of additional data sets necessary for the desired analyses. Users will have to submit any files they want to export from the secure analysis environment to an output control by DZHW staff, ensuring that no identifiable information is exported. Detailed further information on data access can also be found on the DZHW website: https://fdz.dzhw.eu/en/data-usage.

A3.4 Publication date

The SUF is accessible from May 1, 2026. Access to the SUF will be granted for scientific purposes after signing a Data Use Agreement with the data storage provider.
